# 

**Published:** 1894-09

**Authors:** 


### CONTENTS.

PROCEEDINGS.

Alabama Dental Association........ 417
Southern Dental Association........ 431

COMMUNICATIONS.

Cast Aluminum........ 451

SELECTIONS.

A Contribution to Surgery from its Youngest Specialty, Dental Surgery.—The Surgical Engine........ 456
What Shall we Eat?........ 459

COMMENCEMENTS.

Boston Dental College........ 462
East Tennessee Dental Association........ 462

CORRESPONDENCE.

Dr. J. W. Kasbeer........ 463

EDITORIAL.

The National Meetings at Old Point Comfort........ 465
The Wells' Memorial........ 468

